# Psychological experiences of healthcare professionals in Sri Lanka during COVID-19

**DOI:** 10.1186/s40359-021-00526-5

**Published:** 2021-03-24

**Authors:** Bilesha Perera, Bimba Wickramarachchi, Champika Samanmalie, Manjula Hettiarachchi

**Affiliations:** 1 Department of Community Medicine, Faculty of Medicine, University of Ruhuna, Galle, Sri Lanka; 2 Department of Nursing, Faculty of Allied Health Sciences, University of Ruhuna, Galle, Sri Lanka; 3 Ministry of Health and Indigenous Medical Services, Colombo, Sri Lanka; 4 Nuclear Medicine Unit, Faculty of Medicine, University of Ruhuna, Galle, Sri Lanka

**Keywords:** COVID-19, Healthcare professionals, Psychological health, Stigmatization, Self-confidence, Sri Lanka

## Abstract

**Background:**

The upsurge of COVID-19 has caused numerous psycho-social challenges for healthcare professionals because of its ability to spread rapidly in the community and high mortality rate. The seriousness of the disease has led many healthcare professionals plagued by stigma as well as discrimination. In this study, depressive symptomatology, levels of anxiety, and related psychosocial and occupational factors experienced by healthcare professionals in Sri Lanka during COVID -19 were investigated.

**Methods:**

A total of 512 healthcare professionals were surveyed using an online survey. The Generalized Anxiety Disorder 7-item scale, the Center for Epidemiologic Studies Depression Scale-Revised-10, and psychosocial and occupational factors predictive of depression and anxiety were included in the survey questionnaire. Logistic regression determined the factors associated with the presence of depressive symptoms and anxiety.

**Results:**

Results showed that elevated depressive symptoms and anxiety were experienced by 53.3% and 51.3%, respectively, of the participants. No differences in the prevalence of elevated depressive symptoms and anxiety were found between those who were exposed and non-exposed to COVID-19 confirmed or suspected patients. Having a fear of being infected with COVID-19 and spreading it among family members were associated with increased risk of depression. Among those exposed to COVID-19 confirmed or suspected patients, poor occupational safety (OR = 2.06, 95% CI 1.25–3.39), stigmatization (OR = 2.19, 95% CI 1.29–3.72), and heavy workload (OR = 2.45, 95% CI 1.53–3.92) were associated with increased risk of elevated depressive symptoms, whilst poor self-confidence (OR = 2.53, 95% CI 1.56–4.09) and heavy workload (OR = 1.94. 95% CI 1.22–3.12) were associated with increased risk of anxiety.

**Conclusions:**

Fear of being infected and distress caused by fear of spreading it among family members, stigmatization, poor self-confidence, poor occupational safety and heavy workload are vital risk factors that need to be considered in future psychological support services designed for the healthcare professionals in unprecedented outbreaks like COVID-19.

**Supplementary Information:**

The online version contains supplementary material available at 10.1186/s40359-021-00526-5.

## Background

The exponential growth of COVID-19 cases and deaths has caused profound fears and distresses among healthcare professionals in Sri Lanka. In addition, the paucity of information regarding the risk of acquiring the infection, mode of transmission and the non-availability of a clinically proven treatment or a vaccine intensify the psychosocial burden experienced by them during this unprecedented event [1–4]. Social stigma and discriminatory attitudes shown by the community towards frontline healthcare professionals during a serious infectious disease outbreak would eventually make them vulnerable to develop numerous psychological pathologies [5, 6]. Further, such adverse health consequences accumulated over time would hamper their work efficiency and concentration abilities, reducing the quality of their services [7, 8].

The government of Sri Lanka responded quickly to the pandemic by closing the airport in the country, imposing curfew in several districts, and mobilizing people to prepare for a health emergency [9]. Support was sought from the military and police to implement rules and regulations concerning COVID-19 control measures. The Ministry of Health in Sri Lanka consolidated its limited resources and started testing, quarantining, and treating possible cases of COVID-19. The primary healthcare workforce with the help of police and the military, acted vigorously to halt mobility, gatherings of people, and involvement in institutional and home quarantine activities. These actions may have assisted the public as well as healthcare professionals to cope up with the fear and distress associated with this highly contagious virus successfully.

The purpose of this study was to identify the prevalence of depressive symptoms and anxiety and examine how psychosocial and occupational health issues, specific to COVID-19, have influenced healthcare professionals in Sri Lanka. It was hypothesized that adverse consequences of psychosocial and occupational health factors would be associated with elevated levels of depressive symptoms and anxiety.

## Methods

### Participants

A total of 512 healthcare professionals working in health care institutes in Sri Lanka participated in this study. Participation in the study was completely voluntary. Of the total, 108 (21.1%) were male and the mean age was 37.5 years (*SD* = 9.4 years). The majority of participants were nurses (386, 75.4%) followed by medical officers (96, 18.8%) and other allied healthcare professionals (30, 5.8%). A total of 356 participants (69.5%) were working with COVID-19 confirmed or suspected cases at the time of the survey.

### Study setting, design, and data collection instruments

An online survey was conducted from 1 May 2020 to 22 May 2020 using an online survey form (Additional file 1). This online survey form included demographic and psychosocial questions [10–13]. The Generalized Anxiety Disorder 7-item (GAD-7) scale was used to assess anxiety symptoms and the Center for Epidemiologic Studies Depression Scale-Revised-10 (CESD-R-10) was used to assess depressive symptoms. GAD-7 has 7 items and is scored on a four-point Likert scale (0–3) with a total score ranging from 0 to 21 where higher scores reflect greater anxiety [10]. GAD-7 is a reliable and valid scale used in many countries to assess the anxiety level of healthcare workers [11, 12]. CESD-R-10 has 10 questions on a four-point Likert scale (0–3) and the total score ranges from 0 to 30 where higher scores indicate higher depressive symptomatology [13]. CESD-R-10 is a reliable and valid tool to assess depressive symptoms among adults [13, 14]. However, it has been suggested to take higher cutoff points for correctly identifying true positives and true negatives for depressive disorders when using CESD-R-10 as a screening tool [14]. Both scales have been used in online surveys. We used the English versions of both scales. Stigmatization was asked using a single question “Have you ever felt that you are being discriminated or treated badly by the society due to your job as a healthcare professional during this period of COVID -19?”. Four answers were given “Yes, very high,” “Yes, to a considerable extent,” “Yes, but minor,” and “No.” Those who responded with “Yes, very high” or “Yes, to a considerable extent” were further labeled as “stigmatization experienced” cases. Self- confidence was asked using a single statement “I am confident enough to manage this stressful working situation during COVID-19 pandemic.” The given answers were “Not at all,” “Sometimes,” “Mostly,” and “All of the time.” Those who responded with “All of the time” or “Mostly” were further labeled as “high self-confidence” cases. Perceived adequacy of personal protective equipment (PPE) was asked using a single statement “Do you have adequate personal protective equipment to work with COVID-19 patients? The given answers were “Yes”, “No” and “Not applicable.” Occupational safety was asked using a single statement “Do you think that you have been given full occupational safety against COVID-19? Four answers were given “Yes, very much,” “Yes, to a considerable extent,” “Yes, to some extent” and “No, not at all.” Those who responded with “Yes, very much” or “Yes, to a considerable extent” were further labeled as “high occupational safety” cases. Perceived psychological support was asked using a single statement “Have you received any psychological support/counseling to face job responsibilities under this COVID-19 situation? Two answers were given “Yes” and “No.”

### Data collection procedure

Healthcare professionals who were working in healthcare facilities in Sri Lanka were contacted through various means such as personal contacts, social media, information available in telephone directories, and health websites. The health professionals contacted were explained the purpose of the survey and anonymity, emphasizing that their participation was completely voluntary. Their support was requested to disseminate the survey form among other healthcare professionals. The submitted online questionnaires automatically generated excel worksheets and forwarded such data sheets directly to one of the investigators of the research team. Raw data were stored in a excel file (Additional file 2: Data file that contains such data without any identification information).

### Ethics

Ethical approval was obtained from the Ethics Committee, Faculty of Allied Health Sciences, University of Ruhuna, Galle, Sri Lanka. Participation in the survey was on a completely voluntary basis. The survey instrument was anonymous, and the confidentiality of the information collected was assured.

### Data analysis

Data analysis was performed using SPSS (version 25.0). Descriptive data are presented by frequency and percentage. Chi-square test (for categorical variables) and independent sample *t* test (for continuous variables) were used in the data analysis. The significance level was set at α = 0.05 and all tests were two-tailed. Coding of the CESD-R 10 scale was performed according to the CESD analysis guide (0 = rarely or none of the time 1 = some or a little of the time, 2 = occasionally, 3 = all of the time, item 5 and 8 were coded reversely). The total score was calculated through the sum of scores of the 10 items [13]. If any respondent left out answers to more than 2 items, it was removed from the scoring. To identify those with elevated depressive symptoms, the cutoff point was set at ≥ 10. Coding of the GAD-7 was performed according to the GAD-7 analysis guide (0 = not at all, 1 = several days, 2 = over half the days, 3 = nearly every day). Scores of 5, 10, and 15 were taken as the cut-off points for mild, moderate, and severe anxiety, respectively. If the score was 10 or greater, further evaluation was recommended [10].

Percentages of participants with elevated depressive symptoms and with mild, moderate, and severe anxiety were tabulated. Participants were dichotomized based on CESD-R 10 scores and GAD-7 scores (CESD-R 10 score ≥ 10 as 1 and others as 0, and GAD-7 score ≥ 4 as 1 and others as 0). Personal, psychosocial, and work-related factors associated with depressive symptoms and anxieties were determined separately. Significantly associated factors were loaded in the multivariate binary logistic regression models to determine the predictors associated with elevated depressive symptoms and the anxiety of the participants. Odds ratios (OR) and 95% confidence intervals of the ORs were calculated for significant predictors of elevated depressive symptoms and anxiety.

## Results

Out of the total of 512, 356 participants (69.5%) were working with COVID-19 patients or patients suspected to have COVID-19 at the time of the survey, and they were classified as “exposed” individuals. Thus, there were 156 (30.5%) “non-exposed” individuals. Cronbach’s alpha values of the CESD-R10 and GAD-7 were 0.78 and 0.87, respectively. The correlation coefficient between CESD-R10 and GAD-7 was 0.64.

Nearly half of the participants experienced elevated depressive symptoms (53.3%) and mild to severe levels of anxiety (51.3%) (Table 1). Among nurses, 55.7% of the participants experienced elevated depressive symptoms. The corresponding figures for medical officers and other allied healthcare professionals were 43.8% and 53.3%, respectively. The prevalence of mild to severe levels of anxiety was highest among nurses (53.6%), followed by medical officers (45.8%) and other allied healthcare professionals (40.0%).

**Table 1:** Prevalence (number and percent) of elevated depressive symptoms and anxiety among the healthcare professionals by personal and social factors (N = 512)

Variable	Depressive symptoms		Anxiety					
			Mild		Moderate		Severe	
	A	B	A	B	A	B	A	B
Total	193 (54.2%)	80 (51.3%)	151 (42.4%)	65 (41.6%)	20(5.6%)	14 (9.0%)	9 (2.5%)	4 (2.6%)
Gender								
Male	35 (47.3%)	16 (47.1%)	24 (32.4%)	14 (41.2%)	6 (8.1%)	3 (8.8%)	2 (2.7%)	1 (2.9%)
Female	158 (56.0%)	64 (52.5%)	127 (45.0%)	51 (41.8%)	14(5.0%)	11 (9.0%)	7 (2.5%)	3 (2.5%)
Profession								
Medical officer	27 (44.3%)	15 (42.9%)	20 (32.8%)	11 (31.4%)	5 (8.2%)	4 (11.4%)	2 (3.3%)	2 (5.7%)
Nursing officer	162 (56.8%)	53 (52.5%)	127 (44.6%)	47 (46.5%)	15(5.3%)	9 (8.9%)	7 (2.4%)	2 (2.0%)
Other profession	4 (40.0%)	12 (60.0%)	4 (40.0%)	7 (35.0%)	–	1 (5.0%)	–	–
Work experience								
≤ 1 year	20 (42.3%)	8 (80.0%)	13 (43.3%)	4 (40.0%)	4 (13.3%)	2 (20.0%)	1 (3.2%)	–
2–5 years	53 (53.0%)	19 (51.4%)	45 (45.0%)	16(43.2%)	5 (5.0%)	4 (10.8%)	3 (3.0%)	1 (2.7%)
6–9 years	30 (62.5%)	13 (52.0%)	24(50.0%)	13 (52.0%)	5 (10.4%)	1 (4.1%)	1 (2.1%)	1 (4.0%)
≥ 10 years	90 (50.6%)	41 (48.8%)	69 (38.8%)	32 (38.1%)	6 (3.4%)	7 (8.3%)	4 (2.2%)	2 (2.4%)
Worried about your health								
No	22 (42.3%)	9 (45.0%)	14 (26.9%)	8 (40.0%)	1 (1.9%)	1 (5.0%)	1 (1.9%)	1 (5.0%)
Yes, minor	27 (37.0%)	15 (38.5%)	25 (34.2%)	12 (30.8%)	1 (1.4%)	3 (7.7%)	1 (1.4%)	–
Yes, moderate	56 (53.3%)	20 (51.3%)	54 (51.4%)	19 (48.7%)	5 (4.7%)	5 (12.8%)	1 (1.0%)	–
Yes, considerable	51 (70.8%)	23 (62.2%)	32 (44.4%)	16 (43.2%)	9 (12.5%)	2 (5.4%)	2 (2.8%)	2 (5.4%)
Yes, very much	37 (68.5%)	13 (61.9%)	26 (48.1)	10 (47.6%)	4 (7.4%)	3 (14.3%)	4 (7.4%)	1 (4.8%)
Worried about family health								
No	–	6 (40.0%)	4 (50.0%)	7 (46.7%)	–	–	–	1 (4.3%)
Yes, minor	11 (28.9%)	5 (33.3%)	13 (34.2%)	7 (46.7%)	1 (2.6%)	–	1 (2.6%)	–
Yes, moderate	19 (41.3%)	11 (39.3%)	13 (28.3%)	9 (32.1%)	1 (2.2%)	1 (3.6%)	–	–
Yes, considerable	23 (51.1%)	17 (65.4%)	19 (42.2%)	9 (34.6%)	2 (4.4%)	5 (19.2%)	–	1 (3.8%)
Yes, very much	140 (63.9%)	41 (56.9%)	102 (46.2%)	33 (45.8%)	16 (7.2%)	8 (11.4%)	8 (3.6%)	2 (2.9%)
Stigmatization								
No	50 (43.5%)	28 (41.8%)	41 (35.7%)	21 (31.3%)	3 (2.6%)	4 (6.1%)	1 (0.9%)	1 (1.5%)
Yes, minor	72 (52.2%)	39 (60.9%)	58 (42.0%)	30 (46.9%)	13 (9.4%)	5 (7.9%)	3 (2.2%)	2 (3.2%)
Yes, moderate	39 (61.9%)	8 (57.1%)	27 (42.9%)	7 (50.0%)	4 (6.3%)	3 (21.4%)	2 (3.2%)	1 (7.1%)
Yes, very much	32 (80.0%)	5 (45.5%)	25 (62.5%)	7 (63.6%)	–	2 (18.2%)	3 (7.5%)	–
Self-confidence								
Yes all the time	26 (38.8%)	10 (38.5%)	15 (22.4%)	7 (26.9%)	2 (3.0%)	2 (7.7%)	2 (3.0%)	–
Yes, mostly	85 (53.1%)	34 (52.3%)	63 (39.1%)	24 (37.5%)	8 (5.0%)	6 (9.4%)	2 (1.2%)	2 (3.1%)
Sometimes/No	82 (63.6%)	36 (55.4%)	73 (56.7%)	34 (52.3%)	10(10.0%)	6 (12.8%)	5 (4.0%)	2 (4.3%)

A—Working with COVID-19 confirmed or suspected patients (n = 356)

B—Not working with COVID-19 confirmed or suspected patients (n = 156)

No significant differences were found regarding the prevalence of depressive symptomatology or anxiety between the exposed and non-exposed groups. Subgroup analysis by gender and profession showed the same results, though the prevalence rates were slightly higher in women and nurses. The prevalence rates of elevated depressive symptoms were higher among those who had one year or less than one-year work experience (70.0% vs 51.9%, *p* < 0.05) and among those who were having increased fear of being infected with COVID-19 (67.40% vs 45.4%, *p* < 0.05). Further, those who had higher worries about possible COVID-19 contamination by family members due to their occupation, which is a form of work-home interface stress, reported a higher rate of elevated depressive symptoms (61.0% vs 34.7%, *p* < 0.05). Of note, the mean scores of CESD for men and women were 10.7 (SD = 5.4) and 9.2 (SD = 5.7), respectively, indicating gender differences (*p* < 0.05). The mean scores of CESD-R-10 and GAD-7 among nurses were 10.8 (*SD* = 5.3) and 4.9 (*SD* = 3.8), respectively. The corresponding figures for medical officers (CESD-R-10 mean = 9.1 (*SD* = 6.2) and GAD-7 mean = 4.8 (*SD* = 4.6)) and other allied healthcare professionals [CESD-R-10 mean = 8.6 (*SD* = 5.1) and GAD-7 mean = 3.6 (*SD* = 3.3)] were slightly lower than that of nurses.

About 25% of participants were fallen into the category “stigmatization experienced”. Those who were subjected to stigmatization reported higher rates of elevated depressive symptoms and anxiety (Depressive symptoms: 65.6% vs 49.2%, *p* < 0.05, anxiety: 63.3% vs 47.4%, *p* < 0.05). Of the total, 62.1% of participants were fallen into the category “high self-confidence” About one-third of “high self-confidence” participants experienced both elevated depressive symptoms and anxiety while about half of participants who reported having “low” or “no” self-confidence reported the same. Four occupational health issues related to participants’ mental health (occupational safety measures, personal protective equipment, work overload, and psychological support services) were investigated and results are resented in Table 2.

**Table 2:** Prevalence (number and percent) of elevated depressive symptoms and anxiety among the healthcare professionals by health care system related factors (N = 512)

Variable	Depressive symptoms		Anxiety					
			Mild		Moderate		Severe	
	A	B	A	B	A	B	A	B
You have been given occupational safety								
No, not at all	52 (75.4%)	22 (64.7%)	39 (56.5%)	10 (29.4%)	4 (5.8%)	4 (11.8%)	2 (2.9%)	4 (11.8%)
Yes, some what	85 (54.1%)	32 (45.7%)	64 (40.8%)	36 (51.4%)	12 (7.6%)	3 (4.3%)	5 (3.2%)	–
Yes, considerable	42 (46.0%)	19 (50.0%)	35 (38.9%)	13 (34.2%)	3 (3.3%)	4 (10.8%)	2 (2.2%)	–
Yes, very much	14 (35.0%)	7 (50.0%)	13 (32.5%)	6 (42.9%)	1 (2.4%)	3 (21.4%)	–	–
Have you received psychological support								
Yes	31 (53.7%)	8 (57.1%)	23 (39.7%)	3 (21.4%)	3 (5.2%)	–	2 (3.4%)	–
No	162 (54.4%)	72 (50.7%)	128 (42.7%)	62 (44.3%)	17 (5.7%)	14(10.0%)	7 (2.3%)	4 (2.9%)
Work load is heavy								
Yes	118 (66.7%)	32 (65.3%)	90 (50.8%)	23 (46.9%)	12 (6.8%)	4 (8.2%)	6 (3.4%)	2 (4.1%)
No	75 (41.4%)	48 (44.9%)	61 (33.7%)	42 (39.3%)	8 (4.4%)	10 (9.5%)	3 (1.7%)	2 (1.9%)
Provided PPE equipment								
Yes	68 (49.6%)	18 (45.0%)	52 (38.0%)	14 (35.3%)	6 (4.3%)	3 (7.5%)	4 (2.9%)	3 (7.9%)
No	116 (57.1%)	42 (45.0%)	90 (44.3%)	32 (41.0%)	14 (6.9%)	10 (12.8%)	4 (2.0%)	2 (2.6%)
Not applicable	9 (56.3%)	20 (52.6%)	9 (56.3%)	19 (50.0%)	–	1 (2.6%)	1 (6.3%)	2 (5.3%)

A—Working with COVID-19 confirmed or suspected patients (n = 356)

B—Not working with COVID-19 confirmed or suspected patients (n = 156)

Among the participants, the prevalence of elevated depressive symptoms and anxiety were higher among those who reported having poor occupational safety measures in their workplace compared to that of others (Elevated depressive symptoms: 57.9% vs 45.1%, *p* < 0.05; Elevated anxiety levels: 55.5% vs 44.0%, *p* < 0.05) and among those who reported having heavy workload compared to that of others (Elevated depressive symptoms: 66.4% vs 43.0%, *p* < 0.05; Elevated levels of anxiety: 60.6% vs 44.1%, *p* < 0.05) during COVIC-19. Interestingly, of the total, participants who reported stigmatization and “low” or “no” self-confidence were more likely to report heavy workload (Stigmatization: 60.2% vs 38.8, *p* < 0.05, Little or no self-confidence: 54.6% vs 37.7%, *p* < 0.05). Elevated depressive symptoms and elevated levels of anxiety were not associated with the availability of personal protective equipment (PPE) or psychological support services.

Binary logistic regression was performed separately for depressive symptoms and elevated levels of anxiety for the exposed group (n = 356).

Hosmer–Lemeshow goodness of fit test indicated that data would fit the logistic regression model (Table 3). Predictor variables included in the model were gender, work experience, self-confidence, psychological support, occupational safety, stigmatization, availability of PPE, and heavy workload. Gender, work experience, psychological support, and availability of PPE were found to be non-significant predictors of depressive symptoms and elevated levels of anxiety. Poor occupational safety measures (OR = 2.06, 95% CI 1.25–3.39: *p* = 0.004), stigmatization (OR = 2.19, 95% CI 1.29–3.72: *p* = 0.004) and heavy workload (OR = 2.45, 95% CI 1.53–3.92: *p* = 0.001) were associated with elevated depressive symptoms. Self-confidence (OR = 2.53, 95% CI 1.56–4.09: *p* = 0.001) and heavy workload (OR = 1.94, 95% CI 1.22- 3.12: *p* = 0.005) were associated with elevated levels of anxiety. Since there could be more nuanced effects of the predictor variables on the dependent variable that are not apparent because of collapsing the groups when analyzing, multiple linear regression analysis was performed. It was found that in addition to self-confidence and heavy work load, poor occupational safety measures and stigmatization were also significantly associated with elevated levels of anxiety (*p* < 0.05).

**Table 3:** Binary logistic regression; Elevated depressive symptoms and anxiety by gender, work experience and by health and occupational factors (n = 356)

Predictors	Depressive symptoms			Anxiety		
	OR^a^	(95% CI)	*P*	OR^a^	(95% CI)	*P*
Gender						
Male	1			1		
Female	1.531	0.881–2.686	0.130	1.522	0.872–2.655	0.080
Work experience (years)						
Less than 1	1			1		
2–5	0.470	0.196–1.126	0.090	0.527	0.225–1.238	0.142
5–10	0.893	0.322–2.480	0.828	1.196	0.438–3.268	0.727
More than 10	0.546	0.219–1.362	0.194	0.777	0.318–1.900	0.581
Poor self-confidence	1.230	0.756–2.001	0.404	**2.527**	**1.559–4.095**	**0.000**
Poor psychological support	0.966	0.522–1.788	0.911	1.053	0.568–1.952	0.870
Poor occupational safety	**2.063**	**1.255–3.391**	**0.004**	1.485	0.907–2.429	0.116
Stigmatization	**2.197**	**1.295–3.727**	**0.004**	1.351	0.808–2.260	0.251
Inadequate PPE	0.864	0.527–1.416	0.562	1.024	0.626–1.677	0.923
Heavy workload	**2.454**	**1.535–3.925**	**0.000**	**1.948**	**1.221–3.109**	**0.005**
Model summary						
*X*^2c^		8.7			4.2	
H–L goodness-of-fit^b^		0.336			0.835	
−2 Log^d^		442.7			445.5	

The reference category is 1 and bold *p* values denote statistical significance at the *p* < 0.05 level

^a^OR refers to odds ratio

^b^H–L goodness of fit refers to the Hosmer–Lemeshow goodness-of fit statistics

^c^*x*2 refers chi-square

^d^ − 2 log refers − 2 log likely hood

## Discussion

In this study, we examined depressive symptoms, anxiety levels, and psychosocial and occupational factors related to COVID-19 experienced by healthcare professionals in Sri Lanka, a middle-income country in South Asia.

In this sample of 512 healthcare professionals, 53.3% experienced elevated depressive symptoms, 42.2% mild anxiety, 6.6% moderate anxiety, and 2.5% severe anxiety. In line with our results, a similar study (n = 1257) conducted in China in early February 2020 found 50.3% cases of depression and 44.6% cases of anxiety [15]. However, in a study conducted in Singapore (n = 470), only 8.9% of participants were found to be positive for depression and 14.5% for anxiety [16]. The time of the survey, past experiences of working during infectious disease outbreaks, and characteristics of the healthcare systems would possibly explain the observed differences in the rates [7, 17, 18]. Nonetheless, our results showed a substantial worsening in the psychological health of all categories of healthcare professionals in Sri Lanka during this initial period of COVID-19 pandemic. Interestingly, we observed that the mere presence of the pandemic has elevated the fear and distress of all healthcare professionals irrespective of whether they work with COVID-19 positive or suspected cases or not. Thus, future psychological interventions of emergency outbreaks should focus on all categories of healthcare professionals working in healthcare facilities in Sri Lanka.

Women reported more severe symptoms of depression confirming the gendered impact of COVID-19 [15, 18, 19]. It should be noted that in this study, 78.9% were women (86.5% of whom were nurses who are closely working with patients) and most of them had not been received any training related to outbreak emergencies. Further, women in Sri Lanka are entrusted to do informal care within families and their dedication and psychological devotion to family well-being are somewhat exceptional. Such a bond could lead to compassion fatigue [20, 21]. Our results indicated that distress caused by the separation from family members is a strong predictor of depression and anxiety experienced by healthcare professionals. Staying out of their families for long work and quarantine periods would give them profound distress [20, 22, 23]. Further, nursing officers were more likely to work closely and more frequently with risky patients than others, which would result in more emotional exhaustion. Future infectious disease control training and psychological support programs should take into consideration these important factors which are related to the work efficiency of nurses during outbreaks [16]. In addition to gender, work experience was also found to be related to psychological pathologies observed among the participants of this study. Future psychological support services should especially target healthcare professionals who are in their early carrier. It is required to monitor those who confronted with distress and fear because they might be more likely to develop psychiatric morbidity in the future.

Research indicated that the shortage of personal protective equipment (PPE) and other medical equipment reduce the work efficiency of employees due to their increased frustration and insecure feelings that result [24, 25]. Shortage of safety equipment for low-grade healthcare workers was highlighted during COVID-19, and safety procedures and equipment across non-hospital quarantine centers and newly converted centers of COVID-19 are also inadequate [26]. It was observed that the availability of occupational safety measures at workplace would tend to reduce fear and stress among the participants. However, the lack of provision of PPE does not seem to be associated with the development of depressive symptoms or anxiety in healthcare professionals in Sri Lanka. The country has been ranked as one of the top countries in the world for voluntarism [27]. Healthcare professionals are mostly motivated by the nature of Sri Lankan culture to make their own PPE, buy from stores, and prepare their own sanitizers [26], and thereby lower the psychological burden on them. In addition to PPE, testing and containment are essential for preventing occupational health risks related to COVID-19. There have been failures in the social distance practices and screening in healthcare professionals in hospitals across many countries in the world including Sri Lanka [4, 26, 28, 29]. It is necessary to pay careful attention to these safety issues by health authorities in their future outbreak control programs.

In this study, only 14.1% of participants had received psychological support, and no significant differences in the development of depressive symptoms and anxiety were found between those who received and did not receive such psychological support. Material facilities such as transport and sanitation, emergency feeding and protecting, and supporting family members through neighbors in emergencies would provide more than psychological counseling for them to lessen the psychological burden as in the 2004 tsunami incidence [17]. Also, the proliferation of misinformation including sketchy remedies of COVID-19 in social media may have contributed to the increased tension and uncertainty among healthcare professionals [30]. Such perceived stresses must be discussed in psychological counseling sessions designed for healthcare workers. Challenges in regularly disseminating correct information on the spread of the virus and safety measures among healthcare professionals would leave many of them in uncertainty and indecision [31].

Stigmatization has been identified as a major factor associated with psychological pathologies in healthcare workers [32]. Stigmatization is one of the main predictors of elevated depressive symptoms in our sample of healthcare professionals. There were several incidences in Sri Lanka in which COVID -19 patients hid their illness and came to healthcare facilities for the treatment of other illnesses. Such information in social media has fueled fear among the general public and labeled healthcare professionals as asymptomatic carriers of COVID-19 in society. Fear of contamination has downgraded many healthcare professionals and in extreme cases, forced them to leave their rented houses [33]. These emerging issues have resulted in healthcare professionals being isolated and socially rejected [29, 33]. Stigmatization would increase absenteeism from work and lead to increased workloads for the remaining staff. Such a heavy workload makes serious repercussions to the psychological strengths of healthcare professionals, specifically to nurses. To minimize the possible consequences of stigmatization faced by healthcare professionals, it is necessary to recognize the distinctive features of COVID-19 in a social and cultural context [6]. Based on these evidences, those who are stigmatized should be supported through emotional interventions and social policies.

In this study, we found that poor self-confidence was associated with increased depressive symptoms and anxiety. Self-confidence about working abilities and safety conditions in the workplace contributes to increasing work performances in unprecedented events like COVID-19. Nearly 62% of participants in this study reported a moderate to high level of self-confidence, a good indicator of the character of resilience, a personality trait, found in Sri Lankan healthcare professionals. Exposure to civil wars and catastrophic natural events like a tsunami [34] may have cultivated strong psychological traits among healthcare professionals in Sri Lanka to be resilient when challenges occur.

Although there were a few failures, the Sri Lankan government’s preparedness and subsequent actions in controlling the pandemic since its inception should be praised. Precautionary measures practiced by the nurses in the country were highly effective. To date, none of the nurses working in healthcare facilities was infected with COVID -19 due to their work exposure, and there were only 13 deaths in the country due to COVID-19 as of 30 September 2020. This socio-political factor may have contributed to minimizing the psychological burden of healthcare professionals during COVID-19. Future research should explore relationships between these socio-political and ecological factors and psychological well-being in healthcare professionals during infectious disease outbreaks. One limitation of the study is the use of single-item indicators for the variables stigmatization and self-confidence. In addition, the participants who have had experiences of severe stigma due to COVID-19 may have decided not to fill the questionnaire even after they agreed to participate in the study.

## Conclusions

Psychological burden experienced by healthcare professionals in Sri Lanka during COVID-19 is independent of whether they are dealing with COVID-19 suspected or conformed patients, or not. Thus, future psychological support interventions design for catastrophic events like COVID-19 should target the entire healthcare workforce. Fear of being infected with COVID-19 or spreading it among family members and stigmatization were major modifiable risk factors that seem to cause depression and anxiety in healthcare professionals during this COVID-19 pandemic. Higher self-confidence of the healthcare professionals and generosity and volunteerism of Sri Lankan people seem to have contributed to reducing the psychological burden of healthcare professionals. Effort should be put to minimize psychological burden, especially separation distress, in women healthcare professionals during outbreaks. The workload is a factor that seems to intensify depressive symptomatology and anxiety, which may be mediated by self-confidence and stigmatization. Further research is warranted in this area. Dedicated support from the community and armed forces during COVID-19 seem to have contributed to increasing the morale of healthcare professionals in Sri Lanka. Further research is needed in the field of social processes associated with infectious disease control in Sri Lanka.

## Supplementary Information

**Additional file 1.** The online survey form used to collect data regarding psychological experiences and demographic data during the COVID-19 from healthcare professionals.

**Additional file 2.** This excel file contains raw data that were obtained using the online survey form.

## Data Availability

Data file and online survey form are included in this published article as a supplementary file. The raw data sets are available from the corresponding author on reasonable request.

## Abbreviations

COVID-19: Corona virus disease 2019
GAD-7: Generalized Anxiety Disorder 7-item scale
CESD-R-10: Center for Epidemiologic Studies Depression Scale-Revised 10 scale
SPSS: Statistical Package for Social Sciences
*SD*: Standard deviation
PPE: Personal protective equipment
