# Supplementary material for: Psychological experiences of healthcare professionals in Sri Lanka during COVID-19
Source: BMC Psychol. 2021 Mar 24;9:49. doi: 10.1186/s40359-021-00526-5 (PMC7988642; doi:10.1186/s40359-021-00526-5)
Supplement: Supplementary file 1 — Additional file 1. The online survey form used to collect data regarding psychological experiences and demographic data during the COVID-19 from healthcare professionals. [file 40359_2021_526_MOESM1_ESM.pdf]

RESEARCH QUESTIONNIRE

Psychological impact of Covid- 19 on health professionals

Psychological problems related to Covid -19 among health care professional are investigated. This survey will take less than 10 minutes to fill. By filling the questionnaire your consent to participate in this research is given.

Thank you

B. I. Wickramarachchi, University of Ruhuna, Galle.
M. B. C. Samanmali, Ministry of Health, Colombo.

Part 1: Give answers to the followings questions

1. Your age (in years): ………….

2. Gender: Male …… Female ……

3. Your profession
   a. Nursing officer
   b. Medical officer
   c. Other healthcare professional

4. Working experience (years):………

5. Work place
   a. Teaching hospital
   b. General hospital
   c. Base hospital
   d. District hospital
   e. Other

Part 2: Working conditions (Mark the correct answer)

6. Are you currently working with patients infected with Covid-19?
   a. Yes
   b. No

7. Are you currently working with patients suspected with Covid -19
   a. Yes
   b. No

8. Do you think that your current workload is very heavy /unrealistic
   a. Yes
   b. No

9. Do you have adequate personal protective equipment to work with Covid -19 patients?
   a. Yes
   b. No
   c. Not applicable

10. Are you worried about your health due to this Covid-19?
    a. Yes, very much
    b. Yes, to a considerable extent
    c. Yes, but somewhat moderate
    d. Yes. but minor worries
    e. No
11. Are you worried about health of your family members due to your exposure to patients with Covid-19?
    a. Yes, very much
    b. Yes, to a considerable extent
    c. Yes, but somewhat moderate
    d. Yes, but minor worries
    e. No
12. Have you ever felt that you are being discriminated or treated badly by the society due to your job as a health care professional during this period ?
    a. Yes, very much
    b. Yes, to a considerable extent
    c. Yes, but minor
    d. No
13. Do your family members help you to cope up with personal or psychological problems you experience due to Covid-19?
    a. Yes, very much
    b. Yes, to a considerable extent
    c. Yes, to some extent
    d. No, I am helpless
14. Do you think that you have been given full occupational safety against Covid-19?
    a. Yes, very much
    b. Yes, to a considerable extent
    c. Yes, to some extent
    d. No, not at all
15. Have you received any psychological support /counselling to face job responsibilities under this Covid -19 situation?
    a. Yes.
    b. No

Part 3: Your psychological stage over the last week

16. During the past week, I was bothered by things that usually don't bother me.
    a. Rarely or none of the time (less than 1 day)
    b. Some or a little of the time (1-2 days)
    c. Occasionally (3-4 days)
    d. All of the time (5-7 days)
17. During the past week, I had trouble keeping my mind on what I was doing

a. Rarely or none of the time (less than 1 day)
b. Some or a little of the time (1-2 days)
c. Occasionally (3-4 days)
d. All of the time (5-7 days)

18. During the past week, I felt depressed.
    a. Rarely or none of the time (less than 1 day)
    b. Some or a little of the time (1-2 days)
    c. Occasionally (3-4 days)
    d. All of the time (5-7 days)
19. During the past week, I felt that everything I did was on an effort.
    a. Rarely or none of the time (less than 1 day)
    b. Some or a little of the time (1-2 days)
    c. Occasionally (3-4 days)
    d. All of the time (5-7 days)
20. During the past week, I felt hopeful about the future.
    a. Rarely or none of the time (less than 1 day)
    b. Some or a little of the time (1-2 days)
    c. Occasionally (3-4 days)
    d. All of the time (5-7 days)
21. During the past week, I felt fearful.
    a. Rarely or none of the time (less than 1 day)
    b. Some or a little of the time (1-2 days)
    c. Occasionally (3-4 days)
    d. All of the time (5-7 days)
22. During the past week, my sleep was restless
    a. Rarely or none of the time (less than 1 day)
    b. Some or a little of the time (1-2 days)
    c. Occasionally (3-4 days)
    d. All of the time (5-7 days)
23. During the past week, I was happy.
    a. Rarely or none of the time (less than 1 day)
    b. Some or a little of the time (1-2 days)
    c. Occasionally (3-4 days)
    d. All of the time (5-7 days)
24. During the past week, I felt lonely
    a. Rarely or none of the time (less than 1 day)
    b. Some or a little of the time (1-2 days)
    c. Occasionally (3-4 days)
    d. All of the time (5-7 days)
25. During the past week, I could not go "get going” (I could not do things as usual)
    a. Rarely or none of the time (less than 1 day)
    b. Some or a little of the time (1-2 days)
    c. Occasionally (3-4 days)
    d. All of the time (5-7 days)

Part 4: Over the last two weeks (14 days), how often have you been bothered by the followings problems (Mark the correct answer)

26. Feeling nervous, anxious, or on edge
    a. Not at all,
    b. Several days
    c. Over half the day (7 days)
    d. Nearly every day
27. Not been able to sleep or control worrying
    a. Not at all,
    b. Several days
    c. Over half the day (7 days)
    d. Nearly every day
28. Worrying too much about different things
    a. Not at all,
    b. Several days
    c. Over half the day (7 days)
    d. Nearly every day
29. Trouble in relaxing
    a. Not at all,
    b. Several days
    c. Over half the day (7 days)
    d. Nearly every day
30. Being so restless that it's hard to sit still
    a. Not at all,
    b. Several days
    c. Over half the day (7 days)
    d. Nearly every day
31. Becoming easily annoyed or irritable
    a. Not at all,
    b. Several days
    c. Over half the day (7 days)
    d. Nearly every day
32. Feeling afraid, as if something awful might could happen
    a. Not at all,
    b. Several days
    c. Over half the day (7 days)
    d. Nearly every day
33. I am confidence enough to manage stressful working situations during the Covid - 19 pandemic.
    a. Not at all
    b. Some times
    c. Mostly
    d. All the time

Thank you very much for your participation

Please direct your inquires to
Dr M Hettiarachchi, Faculty of Medicine, University of Ruhuna (Tel- 0777792787)
Ms. MBC Samanmali, Ministry of Helath (Tel – 0773662595)
